# Altered Interoceptive Processing in Generalized Anxiety Disorder—A Heartbeat-Evoked Potential Research

**DOI:** 10.3389/fpsyt.2019.00616

**Published:** 2019-09-05

**Authors:** Jiaoyan Pang, Xiaochen Tang, Hui Li, Qiang Hu, Huiru Cui, Lanlan Zhang, Wei Li, Zhuoying Zhu, Jijun Wang, Chunbo Li

**Affiliations:** ^1^Shanghai Key Laboratory of Psychotic Disorders, Shanghai Mental Health Center, Shanghai Jiao Tong University School of Medicine, Shanghai, China; ^2^School of Government, Shanghai University of Political Science and Law, Shanghai, China; ^3^Department of Psychiatry, Suzhou Guangji Hospital, Suzhou, China; ^4^Department of Psychology, Shanghai Mental Health Center, Shanghai Jiao Tong University School of Medicine, Shanghai, China; ^5^Institute of Psychology and Behavioral Science, Shanghai Jiao Tong University, Shanghai, China; ^6^Center for Excellence in Brain Science and Intelligence Technology (CEBSIT), Chinese Academy of Science, Shanghai, China; ^7^Brain Science and Technology Research Center, Shanghai Jiao Tong University, Shanghai, China

**Keywords:** generalized anxiety disorder, eyes-closed (interoception)/eyes-open (exteroception) resting states, heartbeat-evoked brain potential, neural mechanisms, attentional bias

## Abstract

Generalized anxiety disorder (GAD) is one of the most common anxiety disorders. The brain’s dysfunctional processing of interoceptive information is increasingly recognized as an important component of anxiety disorders. However, the neural mechanisms remain insufficiently understood. In the present study, patients with GAD and healthy control participants underwent an eyes-closed (EC) resting state (interoception) and eyes-open (EO) resting state (exteroception) without paying conscious attention to heartbeat. Electrocardiography (ECG) and electroencephalography (EEG) signals were recorded at the same time. The results show that in healthy controls, the heartbeat-evoked brain potential (HEP) was modulated by the conditions, with a significantly higher amplitude under EC than EO, while this was not the case in GAD patients. Further analysis revealed that the dysfunction of HEP modulation in GAD patients may be attributed to excessive interoceptive processing under EO, with a marginally higher HEP in GAD than in the healthy controls. Finally, the right prefrontal HEP amplitude during EC condition was significantly correlated with the severity of the patients’ anxiety symptoms. Our results suggest that altered cortical processing of interoceptive signals may play an important role in the pathophysiology of generalized anxiety disorder.

## Introduction

Generalized anxiety disorder (GAD) is characterized by excessive, uncontrollable, and irrational worry about events or activities (1). This disorder greatly reduces the quality of life of patients and contributes heavily to economic burden (2–4). Interoception refers collectively to the processing of internal bodily stimuli by the nervous system (5). Clinically, patients with GAD always report various interoceptive symptoms, such as elevated heartbeat, sweating, difficulty breathing, feeling tense, and palpitations (6). Dysfunction of interoception is increasingly recognized as to play an important role in the pathophysiology of anxiety and anxiety disorders (5, 7–10). But the neural mechanism of interoceptive processing in GAD is insufficiently studied.

Different methods are used to assess interoception in GAD in clinical studies. In the domain of self-report research, the Body Sensations Questionnaire (11), the Body Vigilance Scale (12), and other questionnaires were frequently used to investigate the patients’ perception of somatic sensations. The patients generally report hypervigilance for physiological information, especially elevated heartbeats (6, 13). From the behavioral indices of interoception, several behavioral paradigms have been developed to assess the accuracy of heartbeat perception. The intraindividual correlation task compute each participant individual correlations between self-report and actual heartbeat (14), tasks based on signal detection theory compare externally generated signals with the rhythm of participant’s own heartbeat (15), and the mental tracking paradigms always recommend the subjects to silently count the heartbeats during several intervals and report the number of heartbeats counted (16). Van der Does et al. (2000) systematically re-analyzed data from studies using the mental tracking procedure and found a subgroup of patients with GAD and panic disorder (PD) who experience frequent or continuous episodes of clinical anxiety symptoms displayed a more accurate heartbeat perception than healthy controls (17). However, results are inconclusive when other task paradigms are used (9). Owing to the fact that some modalities of the body sensations are near or below the level of conscious perception (18, 19), the traditional behavioral methods are unable to differentiate between actual afferent heartbeat signals and the active processes of attention focusing or the participant’s knowledge and beliefs about the heart rate (20–22). Researchers pointed out that with little standardization, the most popular methods for assessing heartbeat perception mentioned above appear to be biased and of questionable validity (23, 24). Physiologic recording studies found that although patients with GAD rated higher on psychic and somatic anxiety symptoms, they showed normal heart rate, skin conductance, and respiration values while at rest and during everyday activities when not feeling tense or anxious (6, 25). The findings suggest that the altered interoception in GAD patients reflects central rather than peripheral response.

Neural responses to heartbeats or heartbeat-evoked brain potential (HEP) is electrocortical potential time-locked to the cardiac R-wave (26) and is interpreted as psychophysiological indicator of cortical processing of cardioceptive signals (27). The HEP can be detected without direct attention or perception to the heartbeat, for example, in the resting state (22, 28, 29), during sleep (30), or when people are focused on external information (20, 31, 32), making it a valuable objective quantitative assessment tool to investigate interoception. A newly published review suggests that when intrinsic limitations (e.g., artifacts) are carefully controlled, the HEP could provide a reliable neural measure for investigating the brain–viscera interactions in diverse mental processes (33). Dysfunction of interoception is increasingly recognized as a pathophysiology of anxiety disorders (5, 9, 10), but the neural mechanism, especially the HEP in GAD, remains insufficiently understood.

It is very important to simultaneously investigate the exteroception in the study of interoception, as the contrast between the two conditions establishes whether the observed effects reflect modulations of general attentional mechanisms or the specific dynamics of internally driven processes (34). Further, the interaction between interoceptive and exteroceptive processing has been proposed as a potential mechanism underlying the generation of perceptual experience (35). The emerging computational neuroscience theories derived from Bayes theorem have integrated interoception and exteroception conceptually (36–38). However, previous interoceptive researches have largely emphasized on interoceptive processing while overlooking exteroceptive conditions (22, 26, 39–42). Therefore, we will establish interoceptive and exteroceptive tasks in the present experimental design.

Previous neuroimaging studies indicate that eyes-open (EO) and eyes-closed (EC) resting states reflect an “exteroceptive” mental state and an “interoceptive” mental state, respectively (43–45). Two studies reported considerably and consistently different brain activation patterns when the environment is dark, with attentional and oculomotor systems (e.g., superior parietal gyrus and frontal eye fields) activated in EO and imagination and multisensory integration systems (e.g., visual, auditory, and somatosensory) activated in EC (43, 46). Another R-fMRI study manipulated both EO/EC and lights on/off, and significant differences between EO and EC in both spontaneous brain activity and functional connectivity were also confirmed (47). Xu et al. further investigated the topological properties of human brain when the eyes were open versus closed. The finding further support the proposition that there are two distinct networks underlying EO and EC resting states. One is the “exteroceptive” network specific to EO for alertness and readiness, composed of the oculomotor system, attentional system, and arousal system. The other is the “interoceptive” network specific to EC for imagination and multiple sensory experiences, which mainly includes the visual system, auditory system, somatosensory system, and part of the default mode network (44). A HEP research of interoception in insomnia used the EO and EC conditions as well (48). Therefore, we established the eyes-closed and eyes-open resting states as interoceptive and exteroceptive conditions, respectively.

In the present study, we assessed heartbeat-evoked brain potentials of patients with GAD and healthy control (HC) subjects during eyes-open and eyes-closed resting states to examine the central nervous system (CNS) representations of afferent signals from the cardiovascular system. Our objective was to investigate whether patients with clinical anxiety show altered HEP responses to cardiovascular information. A recent study manipulated interoceptive and exteroceptive tasks in the same healthy control participants, and the results revealed significantly higher HEP during interoceptive compared with exteroceptive state, reflecting a normal HEP modulation (32). We hypothesized that healthy participants would exhibit normal HEP modulation, whereas the GAD patients would exhibit hypervigilance to interoceptive signals. Specifically, we investigated the following hypotheses: (a) The HEP is significantly higher during EC compared with EO resting state in healthy controls. (b) Patients with GAD may show reduced interoceptive adaptation, and there is no HEP amplitude difference between EC and EO. (c) The altered interoceptive processing in GAD may have originated from their excessive unconscious attentional bias to interoceptive information, showing a higher HEP amplitude in the EO resting state.

## Materials and Methods

### Participants

In total, 40 participants were included in the study, including 15 HC subjects and 25 patients with GAD. Patients were recruited from the psychosomatic outpatient clinic at Shanghai Mental Health Center (SMHC) and Tongji Hospital of Shanghai, China. Patients were diagnosed by one expert clinician based on the *Diagnostic and Statistical Manual of Mental Disorders* (DSM-IV) (49) criteria for GAD. The diagnoses were further checked by two research doctors (HL and QH) using the Chinese version of the Mini-International Neuropsychiatric Interview (MINI) (50). The inclusion criteria for the patients were as follows: Hamilton Anxiety Rating Scale (HAMA) score ≥ 14 (51) and Hamilton Depression Rating Scale (HAMD) score ≤ 14 (52); aged 18–60 years; at least 9 years of education; and medication free for at least 2 weeks. Healthy control subjects were matched for sex, age, and education level and did not meet the DSM-IV criteria for any psychiatric conditions. The exclusion criteria for both the groups were difficulties in communication, severe somatic diseases, alcohol or substance abuse, suicidal tendencies, and pregnant or lactating women. This study was approved by the Research Ethics Committee of Shanghai Mental Health Center, China (SMHC-IRB2012-17). Participants in the HC group were recruited from local communities and were provided with written information about the experiment upon their arrival. All participants provided written informed consent before participating in the experiment.

### Procedure

The subjects first answered a list of questionnaires, including demographic data, HAMA, HAMD, Spielberger State-Trait Anxiety Inventory (STAI-Form Y) (53), and the Chinese version of the Toronto Alexithymia Scale-20 (TAS-20) (54). The Chinese version of the TAS-20 contains 20 items assessing three different aspects of alexithymia: difficulty identifying feelings (DIF), difficulty expressing feelings (DEF), and externally oriented thinking (EOT), all of which are rated on a 5-point Likert scale. This questionnaire shows good internal consistency (Cronbach’s alpha values for DIF, DEF, and EOT, respectively, are 0.645, 0.630, and 0.581 in the control group and 0.739, 0.694, and 0.679 in the clinical group) and test–retest reliability (*r* = 0.782, 0.687, and 0.893 for DIF, DEF, and EOT, respectively, calculated over a 4-week period) (54).

Following administration of these questionnaires, the participants seated upright in a sound-shielded room with the lights turned off in order to resist electromagnetic, visual, and auditory disturbances. This experiment used a within-subjects design, and all participants underwent two sessions of 3-min resting state periods with eyes closed followed by eyes open while electroencephalography (EEG) and electrocardiography (ECG) recordings were obtained. The two conditions were not counterbalanced. In the eyes-closed resting state, we instructed the subjects to sit silently with their eyes closed. After 3-min recording, we instructed them to open their eyes and focus at a black plus sign in the center of a white background on the computer monitor in front of them. During recording, participants were instructed not to think about anything in particular or fall asleep and to not move.

### EEG and ECG Data Recording

EEG signals were recorded using a 64-channel BrainCap (Brain Products GmbH, Germany) in the standard 10–20 system, with the ground electrode placed at FPz. The ECG channels were placed in accordance with the standard lead II configuration. A vertical electro-oculogram (EOG) was recorded supra-orbitally at the left eye, while horizontal EOG was recorded from the right orbital rim. The signals were referenced online to the nose tip and then filtered online with 0.1–200 Hz sampled at 500 Hz. The impedance values of all the electrodes were set below 10 kΩ throughout the EEG acquisition.

### Data Preprocessing

The offline preprocessing was conducted using the EEGLAB toolbox. The R-peak of the ECG channel was first detected using combined adaptive threshold implemented in the FMRIB plug-in (55, 56). The EEG signals were then low-pass filtered at 30 Hz using EEGLAB’s basic FIR filter and were submitted to independent component analysis (ICA) using the runica function. Artifact components like EOG and ECG artifacts were identified through visual inspection of component activations and maps. While analyzing the HEPs, the T-wave-evoked cardiac field artifact (CFA) was a prominent interference factor owing to its superimposition on the HEP (31, 57). Several different approaches have been used to attenuate the CFA, including principal component analysis (PCA) (21), current source density (CSD) (26), ICA (58–60), and the consideration of largely CFA-free HEP intervals (61). Of these, ICA has been shown to remove artifact signals most efficiently (40).

The artifact-free EEG was then segmented into epochs of 1,000 ms time-locked to the R-peak of the ECG with a 200-ms pre-stimulus baseline. The automatic epoch rejection threshold was ± 100 μV, and the bad channels were interpolated using the method of Planar incorporated in EEGLAB. Next, an EEGLAB study was created with two conditions (eyes open vs. eyes closed) across two groups (GAD and HC).

### Statistical Analysis

In order to detect reliable HEP differences with high temporal and spatial resolutions, mass univariate analysis was performed at each time point and electrode between groups and conditions using independent and paired *t*-test, respectively. The multiple-comparisons problem was dealt with by a non-parametric cluster-based permutation procedure (62, 63). For each cluster, its significance probability is calculated using the sum of the *t*-values within that cluster with a Monte Carlo method, which resulted in a permutation distribution of the maximum of cluster-level summed *t*-values. A temporal cluster is considered to be significantly different when the observed cluster-level *p* value is smaller than the critical alpha level of 0.05. Due to the limitation of current EEGLAB STUDY framework, the statistical main effects of a 2 × 2 design could not be directly displayed. With the use of the time window identified by the cluster-based permutation procedure, the mean HEP amplitudes across six electrodes were submitted to three-way repeated-measures analysis of variance (ANOVA) with two within-subject factors (Resting state: EO vs. EC; Electrodes: FPZ, FP2, AF4, AF8, F6, and F8) and one between-subject factor (Group: GAD vs. HC), using a Greenhouse–Geisser correction, where appropriate. Considering the interaction effect, the simple effect of group within the levels of resting states was conducted with the least significant difference (LSD) method for multiple comparisons. Furthermore, the relationship between HEP amplitudes and clinical characteristics was determined using regression analysis that estimated both the strength and direction of the relationship between variables.

The statistical product and service solutions software 17.0 (SPSS, Inc., Chicago, Illinois) was used to compare age, sex, levels of education, HAMA scores, HAMD scores, heart rates, and TAS between groups. Independent sample *t*-tests were used to test differences in continuous variables, while chi-square tests were used for categorical variables. The false discovery rate (FDR) method was taken to deal with the multiple-comparisons problem (64).

## Results

Compared with the healthy control group, the HAMA, HAMA-psychic anxiety, HAMA-somatic anxiety, HAMD, STAI-trait, STAI-state, and TAS-DIF scores were significantly higher in patients with GAD (*p* < 0.005). The age, levels of education, proportion of male and female participants, body mass index (BMI), TAS-DEF scores, and TAS-EOT scores were not significantly different between the two groups. These results are summarized in **Table 1**. The resting state heart rates as indexed by the R-R intervals were not significantly different between the two groups (**Table 2**).

**Table 1:** Demographic and clinical data.

	GAD	HC	*p* value	*p*_FDR_ value
Age (years)	37.70 ± 10.67	39.93 ± 8.94	0.506	0.553
Education (level)	2.62 ± 0.92	2.93 ± 0.88	0.309	0.402
Sex (M/F)	11/17	4/12	0.510	0.553
BMI	21.20 ± 2.54	23.12 ± 3.87	0.076	0.124
HAMA	19.46 ± 6.48	1.13 ± 1.59	<0.001**	<0.001^##^
HAMA-psychic	11.35 ± 4.14	1.00 ± 1.59	<0.001**	<0.001^##^
HAMA-somatic	8.12 ± 3.10	0.13 ± 0.34	<0.001**	<0.001^##^
HAMD	10.62 ± 5.29	1.19 ± 1.47	<0.001**	<0.001^##^
STAI-trait	51.71 ± 11.15	38.50 ± 7.09	<0.001**	<0.001^##^
STAI-state	52.18 ± 13.79	34.79 ± 8.26	<0.001**	<0.001^##^
TAS-DIF	22.13 ± 7.01	15.40 ± 5.64	0.004*	0.007^#^
TAS-DEF	14.52 ± 3.82	13.00 ± 3.05	0.204	0.294
TAS-EOT	20.00 ± 3.42	19.73 ± 3.51	0.818	0.818

GAD, generalized anxiety disorder; HC, healthy control; HAMA, Hamilton Anxiety Rating Scale; HAMD, Hamilton Depression Rating Scale; BMI, body mass index; STAI, State-Trait Anxiety Inventory; TAS, Toronto Alexithymia Scale; DIF, difficulty identifying feelings; DEF, difficulty expressing feelings; EOT, externally oriented thinking.

*p < 0.05.

**p < 0.001.

^##^p_FDR_ < 0.001.

**Table 2:** Measures of heartbeats.

	GAD		HC		Main effect		Interaction
	EO	EC	EO	EC	Resting state	Group	Resting state*Group
R-R	806.68 ± 144.34	824.68 ± 122.42	847.01 ± 113.77	840.61 ± 100.98	0.602	0.474	0.275

GAD, generalized anxiety disorder; HC, healthy control; EO, eyes-open; EC, eyes-closed; R-R, inter-beat (RR) interval.

**Figure 1:**
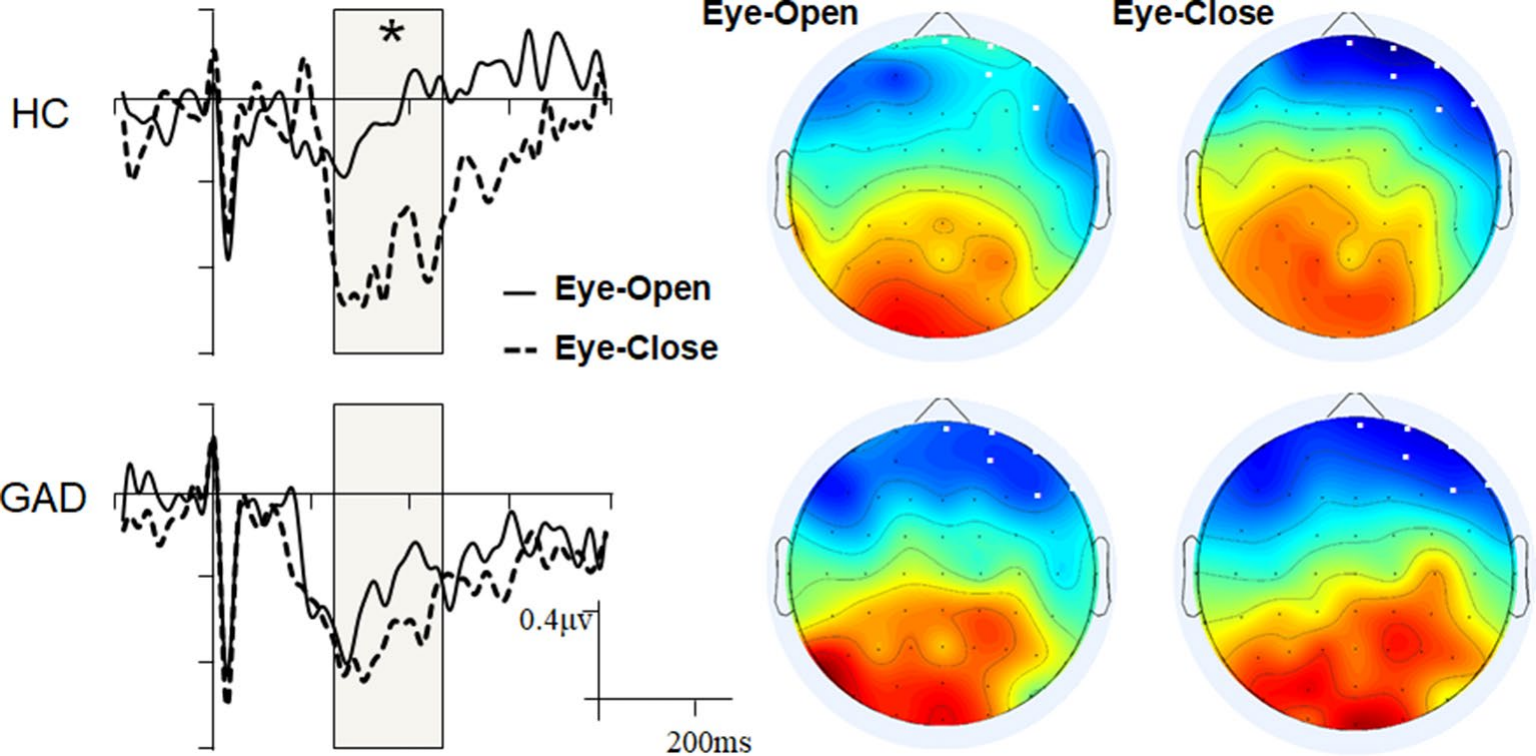
Grand event-related potentials (ERPs) and scalp topology. The ERP waveforms were averaged across the six selected electrodes with the highlighted significant time window.

Mass univariate analyses were used to explore differences between the groups or conditions (resting states). While we found no significant group difference within the EO and EC states, we observed significant interoceptive–exteroceptive state modulation in the HC group with latencies ranging from 240 to 460 ms, but not in the GAD group (**Figure 1**). The HEP modulation was a negative waveform difference over the right prefrontal areas in the control group, which was more negative in the EC state than in the EO state. The main effect of Resting state and the Resting state * Group interaction effect were statistically significant (*F*_1,38_ = 30.750, *p* < 0.001, and *F*_1,38_ = 8.715, *p* = 0.005, respectively). The GAD group showed a marginally significant higher negative HEP amplitude than the HC group in the EO state (GAD: −0.524 vs. HC: −0.220, *p* = 0.053), but not in the EC state (GAD: −0.706 vs. HC: −0.816, *p* = 0.434).

**Figure 2:**
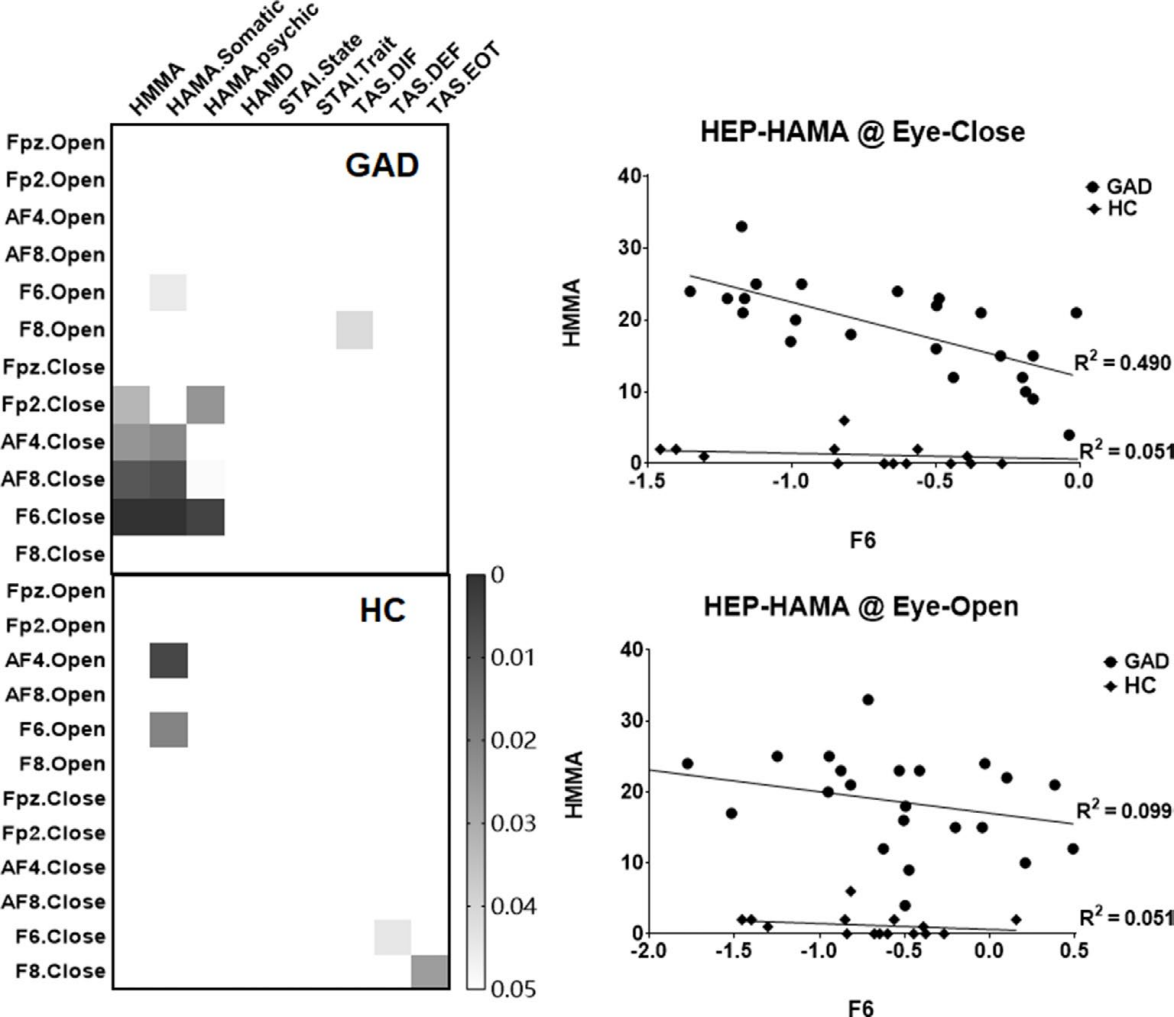
Heat maps and scatter plots of the correlation analyses. The left panel highlights the significant correlation (p < 0.05, uncorrected), and the right panel illustrates the relationship between the mean amplitude of HEP (at F6 electrode across four conditions) and the HAMA scores.

In order to examine the clinical relevance of our HEP findings, exploratory correlation analyses were conducted between the mean amplitudes of the right prefrontal HEP component and the various questionnaire scores. We found a significant association between the HEP amplitude and anxiety symptoms in the GAD group under the EC state (**Figure 2**), which indicates that higher negative amplitudes of the HEP component reflect higher levels of experienced anxiety.

By resampling the observed *p* value matrix (24 * 9) for 5,000 times, the 99.9th percentile in the resulting distribution of the maximum of cluster size is 8.5 (less than the observed cluster size of 9), which confirmed that there was a statistically significant correlation between HEP amplitudes and HAMA symptoms. Furthermore, by correction multiple comparisons with FDR method, the correlation between HEP amplitude of F6 under EC condition with HAMA and HAMA-Somatic reached statistical significant (*p* = 0.028 and *p* = 0.028, respectively). The difference of *r* values between the two groups has a trend toward significance (*z* = −1.7425, *p* = 0.0814), and the statistical power could be improved by the increased number of HC compared with GAD (HC: 15- > 23, *z* = −2.0121, *p* value = 0.0442).

## Discussion

The current study investigated and compared the cortical interoceptive processing in patients with GAD and healthy control subjects. The cortical processing of interoceptive signals was assessed quantitatively by measuring the amplitude of HEP under the eyes-open and eyes-closed resting states. The mass univariate analyses revealed a significantly more negative HEP amplitude in EC than EO condition with the latencies ranging from 240 to 460 ms in healthy control participants, but not in the GAD group. Further ANOVA analysis revealed that the GAD group showed marginally significant higher negative HEP amplitude than the HC group in the EO state (*p* = 0.053), but not in the EC state. The patients’ HEP amplitude under interoceptive state is correlated with their anxiety scores.

In the present study, the demographic variables were carefully matched between the two groups. Except for the difference of anxiety symptoms as measured by the HAMA and STAI questionnaires, the GAD patients exhibited significant stronger depressive symptoms. Previous studies showed that the HEP amplitude measured in a group of major depression patients recorded during a heartbeat counting interoceptive task was lower than in healthy controls (40). Because our result in GAD is of the opposite direction, therefore, we believe the present higher HEP cannot be attributed to the comorbid depressive symptomatology. We found that the total TAS score and the DIF score are significantly higher in patients with GAD than in healthy control subjects. This result is consistent with the previous study, which reported heightened sensitivity for interoceptive signals combined with a difficulty in attributing these sensations to emotions (alexithymia), which would increase an individual’s vulnerability to anxiety (65). The ECG result as revealed by the R-R interval showed no difference between groups and conditions, a finding consistent with results from previous studies (6, 25), suggesting that the HEP difference reflects neural activity responding to heartbeats rather than peripheral differences.

A higher HEP component in the fronto-central electrodes within similar time windows has been reported repeatedly in previous studies (20, 41, 66, 67). The present result is consistent with our hypothesis that the HEP is significantly higher during EC compared with EO resting state in healthy controls, whereas patients with anxiety disorder show reduced interoceptive adaptation, with no difference in HEP amplitude between EC and EO conditions. In the eyes-closed resting state, normal interoceptive processing should be activated (43–45), and the eyes-open resting condition would interfere with interoception by increasing vigilance and alerting the subjects to exteroceptive changes. Therefore, more negative waveforms over the right prefrontal areas in HC participants in the EC state (compared with the EO state) reflect adaptive cortical processing of interoceptive signals, with higher HEPs in response to the interoceptive task and lower HEPs in response to the exteroceptive task. This result in healthy controls is to some extent consistent with the research of Petzschner F. H., et al. (2019), in which the HEP is significantly higher during interoceptive compared with exteroceptive attention in healthy controls (32). In their experimental design, participants were instructed to pay conscious attention to interoception or exteroception, whereas in our study, the HEP was obtained without requiring subjects to perceive their heartbeats. Contrary to their conclusion that the HEP is modulated by pure attention, the HEP modulation in the present study should be associated with unconscious/subconscious attention or cardiac interoceptive processing.

We address the origin of deficient interoception adaptation in generalized anxiety disorder by further analysis of the interaction between the groups and resting states. In agreement with our hypothesis, while anxiety disorders elicited a marginally higher HEP amplitude during the EO state, there is no group-wise difference in the HEP amplitude in the EC state. Higher HEP amplitudes during the EO state in patients with GAD may reflect excessive cortical processing of afferent cardiac signals when external visual information needs to be processed. This phenomenon may be attributed to unconscious attentional bias toward anxiety-related body sensations in GAD, similar to the increased interoceptive processing in insomnia (ID) (48), obsessive-compulsive disorder (OCD) (68), and the social anxiety disorder (69). Insomnia is characterized with physiological and cortical hyperarousal, and the higher amplitude of late HEP component in ID than controls at frontal electrodes during EC and EO resting states was interpreted as a possible attentive processing of afferent stimuli (48). Patients with OCD exhibit greater HEP amplitude modulation during cardiac interoception, indicating that hyperactive monitoring in OCD extends to the sensing of internal bodily signals (68). Enhanced HEP amplitude during false feedback of accelerated heart rate in a study of social anxiety is consistent with the theoretical prediction of increased self-focus driven by concerns of somatic symptoms (69). The reduced HEP amplitudes indicative of deficient attentive processing of bodily experiences in patients with depression and depersonalization/derealization disorder (DPD) (40, 42, 70) support our results as well. The authors speculated that the reduced HEP amplitude in depressed patients reveal the altered body perception or reduced interoceptive awareness (40). Results from previous studies suggest that patients with DPD do not exhibit higher HEPs during the heartbeat perception task than during rest, indicating deficient attentive processing of their actual bodily experiences (42). To sum up, the HEP as quantitative read-outs of interoceptive information processing could become valuable diagnostic tools for detecting aberrant attention to interosensations in GAD patients who show overly salient sensory signals from the body (32).

We also investigated whether interoceptive responses such as HEP is associated with clinical symptoms of anxiety. Our results demonstrate a significant correlation between clinical anxiety symptom scores and the HEP amplitude over the right prefrontal areas during the eyes-closed resting state in patients with GAD. This result is similar to the insomnia study, which found that The average amplitude of the late frontal HEP component during EC correlated significantly with the subject’s sleep complaints (48). Therefore, we speculate the HEP amplitude under interoceptive conditions may be an objective quantitative marker of the GAD patients’ anxiety level. A functional magnetic resonance imaging study supports our conclusion by reporting that the cingulo-opercular task control network connectivity might represent excessive attention to interoceptive information, and abnormal activation patterns in these areas might cause an individual’s bodily signals to be unreasonably amplified, making them feel much anxious (71).

## Limitations

A limitation of the present study is that the block sequence of eyes-open and eyes-closed resting states was not counterbalanced across subjects. Thus, our findings may not be generalized to other situations, such as the transition from the eyes-open state to the eyes-closed state. Future study will be needed to investigate the EO-to-EC transition. Second, the relatively small sample size of our study may weaken the results’ reproducibility. Thus, future multi-site studies with bigger sample sizes are needed to establish our results.

## Conclusions

To our knowledge, this is the first objective and quantitative HEP assessment of cortical interoceptive processing in generalized anxiety disorder. In conclusion, the current findings show that interoceptive and exteroceptive state transition fails to modulate the HEP amplitude in GAD, suggesting that GAD is characterized by deficient adaptation to interoceptive signals. Further analysis reveals that the deficiency in adaptation may result from exaggerated attention bias assigned to afferent bodily signals when external attention is required. HEP assessment under interoception and exteroception conditions may provide a valuable paradigm to explore the pathophysiology of generalized anxiety disorders.

## Ethics Statement

This study was carried out in accordance with the recommendations of Ethical Conduct for Research Involving Humans (the TCPS-2), Research Ethics Boards at Shanghai Mental Health Center of Shanghai Jiao Tong University with written informed consent from all subjects. All subjects gave written informed consent in accordance with the Declaration of Helsinki. The protocol was approved by the Research Ethics Committee at Shanghai Mental Health Center.

## Author Contributions

JP, XT, CL, and JW contributed to the conception and design of the study. HL, QH, WL, LZ, and HC did the experiment and data acquisition. JP and XT performed the statistical analysis. JP, XT, and ZZ wrote the first draft of the manuscript. JP, XT, JW, CL, and ZZ revised the manuscript critically for important intellectual contest. All authors contributed to manuscript revision and have read and approved the submitted version.

## Funding

This work was supported by the National Natural Science Foundation of China (81071098) to CL, Shanghai Science and Technology Committee (16JC1420200) to JW, Shanghai Science and Technology Committee (16411965000) to HL, Shanghai Jiao Tong University Foundation (YG2016MS37) to HL, Shanghai Municipal Natural Science Foundation (18ZR1432600) to HC, and Shanghai Science and Technology Committee (18411952400) to HC.

## Conflict of Interest Statement

The authors declare that the research was conducted in the absence of any commercial or financial relationships that could be construed as a potential conflict of interest.

## Abbreviations

EC, eyes-closed; EO, eyes-open; HC, healthy control.
