# Supplementary material for: Altered Interoceptive Processing in Generalized Anxiety Disorder—A Heartbeat-Evoked Potential Research
Source: Front Psychiatry. 2019 Sep 5;10:616. doi: 10.3389/fpsyt.2019.00616 (PMC6739601; doi:10.3389/fpsyt.2019.00616)
Supplement: Supplementary file 1 [file Table_1.pdf]

2 - Since adjacent electrodes usually have somewhat correlated signals, the averaged 6-electrode signal is probably a better candidate for regression analysis using the Anxiety symptoms as predictors of the HEP.

The correlation result of averaged HEP amplitudes across selected electrodes is shown as follows (Table S1), which also found strong association between HEP amplitude under EC condition with anxiety symptoms. Unfortunately, none of these p values survived FDR procedure. Thus, we choose to provide correlation results of channels as stated in the previous comment.

Table S1.

		HAMA	HAMA-psychic	HAMA-somatic	HAMD	STAI-trait	STAI-state	TAS-DIF	TAS-DEF	TAS-EOT
GAD	HEP.Close	0.007	0.011	0.025	0.575	0.645	0.45	0.307	0.416	0.243
	HEP.Open	0.238	0.05	0.721	0.359	0.162	0.214	0.946	0.467	0.068
HC	HEP.Close	0.624	0.542	0.724	0.373	0.628	0.935	0.416	0.092	0.156
	HEP.Open	0.347	0.058	0.591	0.427	0.938	0.475	0.982	0.601	0.386
